# Supplementary material for: The Serum Cell-Free microRNA Expression Profile in MCTD, SLE, SSc, and RA Patients
Source: J Clin Med. 2020 Jan 7;9(1):161. doi: 10.3390/jcm9010161 (PMC7020053; doi:10.3390/jcm9010161)
Supplement: Supplementary file 1 [file jcm-09-00161-s001.pdf]

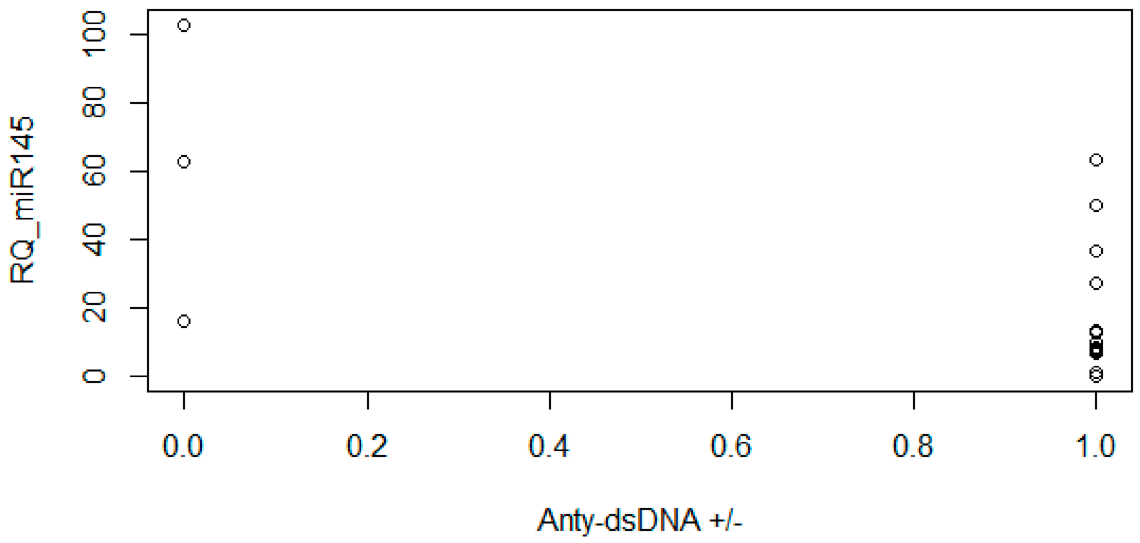

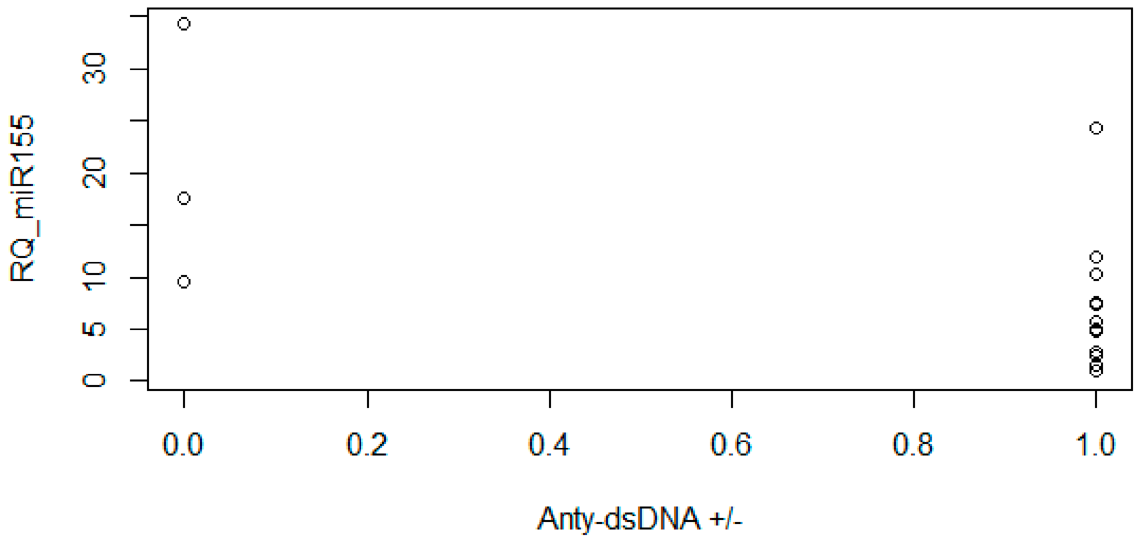

Figure S1. Negative Correlation between miRNAs: miR145 and miR155 serum expression levels and Anty-dsDNA antibody presence in SLE patients.
